# Raspberry-Like Polysilsesquioxane Particles with Hollow-Spheres-on-Sphere Structure: Rational Design, Controllable Synthesis, and Catalytic Application

**DOI:** 10.3390/polym11081350

**Published:** 2019-08-14

**Authors:** Jian Li, Fuping Dong, Liangyu Lu, Hongwei Li, Yuzhu Xiong, Chang-Sik Ha

**Affiliations:** 1 Department of Polymer Materials and Engineering, Guizhou University, Guiyang 550025, China; 2 Department of Polymer Science and Engineering, Pusan National University, Busan 46241, Korea

**Keywords:** hierarchical structure, polysilsesquioxanes, nanocapsules-on-microspheres, catalyst

## Abstract

Raspberry-like hollow-spheres-on-sphere (HSOS) particles with reactive surfaces, uniform sizes and monodisperse properties were rational designed and fabricated to immobilize gold nanoparticles for the catalytic reduction of 4-nitrophenol. HSOS polysilsesquioxane (PSQ) particles were constructed by an organic alkali catalyzed sol-gel process from trialkoxysilane precursors with stabilized polystyrene (PS) nanoparticles as both a sacrifice template and a Pickering emulsifier. The PSQ particles were fabricated in an ice bath with methyltrimethoxysilane and mercaptopropyltrimethoxysiane as a co-precursor, tetramethylammonium hydroxide (TMAH) as a catalyst, polyvinylpyrrolidone (PVP) and sodium lignosulfonat as co-stabilizers and PS latex as a hard template. The formation mechanism of the hierarchical particles was investigated in detail by the time study through imaging the particles at regular time intervals during the reaction process. Various effect factors on the morphology were studied systematically which showed that the precursor composition, the content of PS, TMAH and PVP are the most important factors. The hierarchical structure combined with the mercaptopropyl groups on both the surface and the skeleton to make it possible to adsorb guest molecules. Au nanoparticles were immobilized on the particles for the catalytic reduction of 4-nitrophenol to 4-aminophenol. The unique PSQ colloids with hollow-spheres-on-sphere extended the family of the hierarchical structures and has shown the potential applications in separations, drug delivery and heterogeneous catalysts.

## 1. Introduction

Hierarchical particles with dual-scaled micro-/nanostructures have attracted considerable attention in the field of catalyst, energy, separation, and biomedicine considering their special topography and surface chemistry [1,2,3,4,5,6,7,8]. Micro-/nano-hierarchical particles can not only effectively avoid agglomeration of nanoparticles and easily be handled/recycled in comparison with nanoparticles but also produce porous structures during the assembling process [9,10]. Among hierarchical materials, raspberry-like particles with large central spheres surrounded by small spheres have gained increasing attention given their unique morphology, rough surfaces, large surface areas, and adjustable surface chemistry [11,12,13,14]. These raspberry-like nanoparticles have been investigated and utilized in versatile applications, such as the building blocks for superhydrophobic surfaces, delivery of drugs, optical materials, and as a heterogeneous catalyst [15,16].

Versatile approaches have been developed to fabricate raspberry-like composite particles; these approaches include assembling as-prepared small particles onto as-prepared large particles [17,18], in situ synthesis of small particles on an as-prepared large particle core [19,20,21], in situ synthesis of a large particle core with as-prepared small particles as a Pickering emulsifier [22,23,24], and one-pot sol–gel process with two-stage nucleation of silane precursors [25,26,27]. Ming et al. firstly prepared epoxy-functionalized silica particles of about 700 nm and amine-functionalized silica particles of about 70 nm, then covalently grafted the small particles onto the big ones via the reaction between epoxy and amine groups to form the raspberry-like silica particles with very uniform sizes [17]. In our previous work, microsized polystyrene (PS) spheres have been pre-synthesized as core particles and ethyl-functionalized organic silica nanoparticles were grown on the cores to form raspberry-like composite particles with superhydrophobic properties [19]. Wu et al. prepared raspberry-like particles with poly(methyl methacrylate) as core and nanosilica particles as a shell from the polymerization of the positively charged monomer in the presence of the as-prepared negatively charged nanosilicas [23]. Zhang et al. fabricated sphere-on-sphere (SOS) silica microspheres with a single layer of nanospheres coating on the surface with 3-mercaptopropyltrimethoxysilane (MPTMS) as a precursor [25]. Despite all these successes in the construction of raspberry-like structures, the fabrication of particles by integrating nanocapsules or hollow nanospheres with dense microspheres remains a challenge. Hollow-spheres-on-sphere (HSOS) particles not only have excellent properties of normal dense-sphere-on-sphere particles but also offer unique free volume as carriers for guest molecules.

**Scheme 1:**
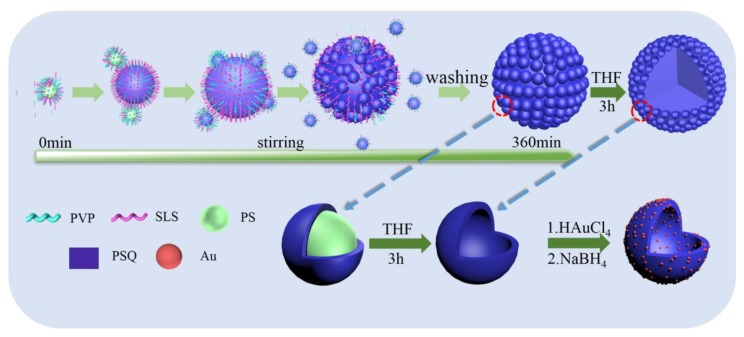
Fabrication process of HSOS particles and Au/HSOS particles.

In the present paper, we report novel hierarchical raspberry-like polysilsesquioxane (PSQ) particles with a HSOS structure via a facile and robust process (Scheme 1). To the best of our knowledge, this work is the first report on the fabrication of particles with a HSOS structure. The particles combine the active mercapto (–SH) groups on the surface with a special topographic feature, that is, the inner microsphere is surrounded by hollow nanospheres. In Scheme 1, the fabrication approach involves hydrolysis–polycondensation of methyltrimethoxysilane (MTMS) and 3-mercaptopropyltrimethoxysilane (SHTMS) trialkoxysilane mixed precursors with tetramethylammonium hydroxide (TMAH) as the catalyst. In addition, polyvinylpyrrolidone (PVP) and sodium lignosulfonate (SLS) co-stabilized polystyrene (PS) latex as the solid Pickering emulsifier. An aqueous solution mixture of PVP, SLS, and a PS suspension was first prepared. Second, TMAH and silane precursors were injected into the suspension sequentially. The reaction was stirred for 6 h in an ice bath. Finally, the HSOS particles were obtained after separation, treated with tetrahydrofuran (THF), and freeze-dried. Even though there are some highly porous/hollow nanoparticles to immobilize Au nanoparticles [28,29], our micro/nano hierarchical structured particles demonstrated the unique advantages such as they could prevent the aggregation of Au nanoparticles and be easily recycled. The new HSOS particles show the high potential for the application in the field of separation, drug delivery and as a heterogeneous catalyst.

## 2. Materials and Methods

### 2.1. Materials

3-mercaptopropyltrimethoxysilane (SHTMS, 95%), methyltrimethoxysilane (MTMS, 98%), styrene (99%), tetramethylammonium hydroxide (TMAH, 25% aqueous solution), sodium lignosulfonate (SLS), polyvinylpyrrolidone (PVP, Mw 40,000), tetrachloroauric acid (HAuCl4, ≥99.9%), potassium persulfate (KPS, 99.9%), 4-nitrophenol (4-NP, 99%) and tetrahydrofuran (THF, 99%) were purchased from Aladdin (Shanghai, China). Sodium borohydride (NaBH4, 98%) was purchased by Tianjin Kermel Chemical Reagent Co., Ltd, Tianjin, China. The water used throughout the experiment was distilled water (~17 MΩ) produced by the Milli-Q water system.

### 2.2. Experimental Methods

#### 2.2.1. Fabrication of HSOS Particles

Polystyrene latexes were prepared by the method as described elsewhere [30]. Typically, 0.01 g of sodium lignosulfonate and 0.02 g of polyvinylpyrrolidone were dissolved in 18 mL of distilled water with stirring in an ice bath until a light yellow-brown transparent solution was obtained. Then, 2 g of the as-prepared PS suspension (0.2 g solid content) was added into the mixture with stirring to make them well dispersed to obtain a milky white suspension. Finally, 0.1 mL of tetramethylammonium hydroxide was added immediately followed by the dropwise injection of the mixed silane mixture containing 15 mmol of methytrimethoxysilane and 1.5 mmol of 3-mercaptopropyltrimethoxysilane. The reaction was carried out with stirring in an ice bath for 6 h and white SOS particles were obtained after washing with water by centrifugation and freeze-drying. The final HSOS particles were obtained by soaking the dry samples in THF for 3 h, washing with THF and freeze-drying.

#### 2.2.2. Au/HSOS Hybrid Particles

The hybrid composites were fabricated through an aqueous impregnation process with HSOS particles as support and HAuCl_4_ as the gold source. 10 mL freshly prepared HAuCl_4_·3H_2_O solution (2 mM) was added to 20 g of HSOS particle suspensions (0.02 g solid containing) under magnetic stirring at room temperature. After stirring for 12 h, 10 mL freshly prepared NaBH_4_ (8 mM) was added into the mixture under stirring at room temperature. After stirring for 3 h, the product was collected by centrifugation and dried at 60 °C overnight in a vacuum oven for further use.

#### 2.2.3. Catalytic Reduction of 4-Nitrophenol

Au/HSOS hybrid composite aqueous suspension (5 mL, 0.4 g/L) was mixed with NaBH4 aqueous solution (1.5 mL, 0.07 mM) under stirring at room temperature. Then, 4-nitrophenol (1.5 mL, 0.034 M) was added to the mixture, which was stirred until the bright yellow gradually changed to colorless. The reaction progress was monitored by measuring UV-vis absorption spectra of the mixture. To study the recycle of the catalyst, the particles were centrifuged after reaction, and the clear supernatant liquid was decanted carefully. The catalyst was washed thoroughly with water, followed by drying at 60 °C for 6 h in a vacuum oven. Then, the catalyst was reused for subsequent recycles under the same reaction conditions.

### 2.3. Characterization

The morphology of all samples was observed by scanning electron microscopy (SEM) using a FEI-SEM system (FEI Helios Nanolab 600i, Hillsboro, USA)) operating at 5 kV without specimen tilt. The samples were dispersed in water and treated in an ultrasonic bath for 10 min. Then the suspension was cast on a small piece of silicon wafer and dried in an air atmosphere overnight. Before measurement, a thin gold film was sprayed on samples. For the particle size estimation, over 100 particles on the SEM images were averaged. Transmission electron microscopy (TEM) images were taken using a FEI Tecnai G2F30 electron microscope (Hillsboro, USA) operating at 200 kV without specimen tilt. The diluted aqueous suspension of the sample was dropped on a carbon coated copper TEM grid (300 mesh, Beijing Zhongjinkeyi Technology Co., Ltd., China) and dried in an air atmosphere overnight for measurement. Fourier transform infrared spectroscopy (FTIR) of KBr powder-pressed pellets with about 1 wt.% of the sample was recorded on a Perkin–Elmer Spectrum GX-spectrophotometer (Waltham, USA) with spectral resolution of 1 cm^−1^ and scan number of 32. X-ray diffraction (XRD) pattern of the sample was recorded using a Philips diffractometer with a Geiger counter (Eindhoven, The Netherlands). The X-ray tube was operated at 40 kV and 30 mA (Cu Kα radiation with Ni filter, λ = 1.5406 Å). Scans were made from 5° to 60° (2θ) at the speed of 1°/min. The ^29^Si NMR spectrum was obtained on a Varian Inc., 400 MHz UNITY INOVA spectrometer (Palo Alto, CA) at room temperature with the resonance frequencies of 79.5 MHz, a magic-angle spinning at 5 kHz, 90° pulse length of 6.5 μs and a repetitions delay of 60 s. Nitrogen adsorption to desorption measurements (ASAP 2046, Micromeritics, USA) at 77 K were performed on the particles to assess their Brunauer–Emmett Teller (BET) surface areas and pore size. Inductively Coupled Plasma (ICP) analysis was performed using a PerkinElmer Nexion 300 (Baesweiler, Germany). UV-vis spectra were recorded using the Evolution 201 (Thermo, America) UV-Vis spectrophotometer with 1 cm quartz cuvettes.

## 3. Results

### 3.1. Characterization of HSOS Particles

**Figure 1:**
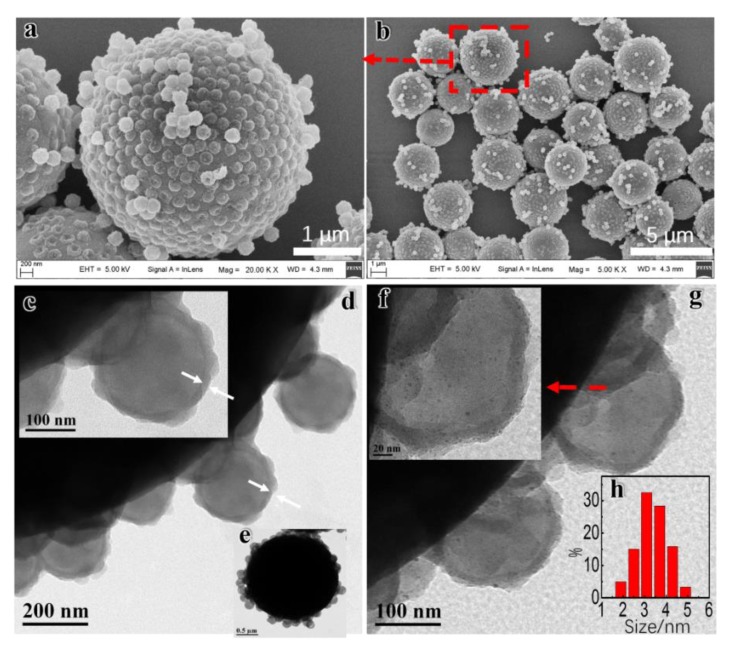
SEM images of hollow-spheres-on-sphere (HSOS) particles at (**a**) high and (**b**) low magnification; TEM images of HSOS particles at (**c**–**e**) different magnification which show hollow nanospheres with 200 nm core and 25 nm shell; TEM images of an Au/HSOS particle at (**f**,**g**) different magnification and (**h**) the particle size distribution of Au nanoparticles.

**Figure 2:**
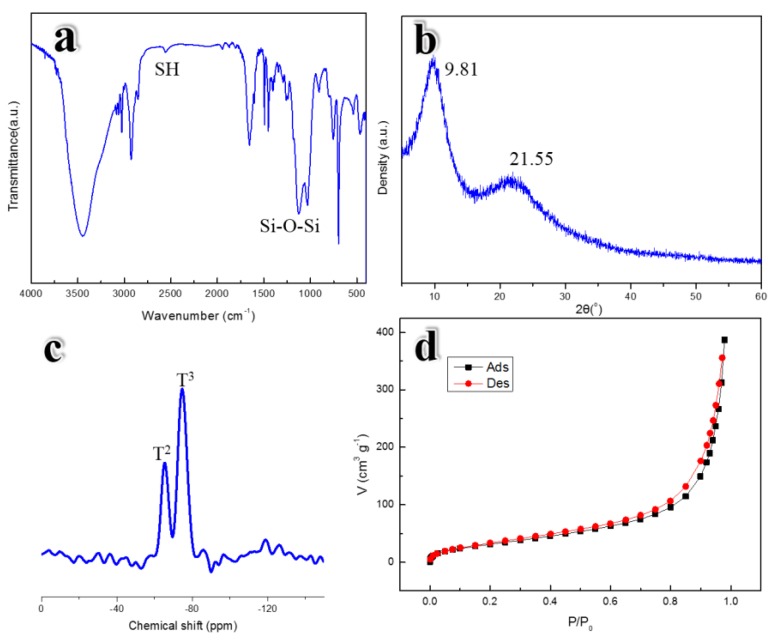
Characterization of HSOS particles. (**a**) FTIR spectrum, (**b**) XRD pattern, (**c**) 29Si NMR spectrum and (**d**) N2 adsorption–desorption isotherm.

The scanning electron microscopy (SEM) images illustrated in Figure 1a show that a large microsphere was found coated with nanospheres with a diameter of 250 nm. Several broken nanocapsules also could be observed which proved the hollow structure of the nanospheres. The average particle size was around 2.4 µm, as estimated by Figure 1b. The transmission electron microscopy (TEM) images depicted in Figure 1c–e clearly exhibit an HSOS structure. The dense microspheres were surrounded by hollow nanospheres with hollow cores of nearly 200 nm in diameter and a shell thickness of approximately 25 nm. In the Fourier transform infrared (FTIR) spectrum of the HSOS particles (Figure 2a), the strong absorption peaks at 1123 and 1026 cm^−1^ represent Si–O–Si structures of PSQs [31]. The peaks at 2555 cm^−1^ for the SH group and 780 cm^−1^ for the SiCH_3_ group were observed in the HSOS particles, thereby demonstrating the successful formation of mercaptopropyl-functionalized PSQs [32]. In Figure 2b, the X-ray diffraction pattern of the HSOS particles showed two peaks. The first sharp peak at 9.81° was attributed to the intermolecular spacing of the silsesquioxane components. Moreover, the second broad peak at 21.55°, which was believed to be related to the ladder-like double chain on PSQs, was assigned to the intramolecular siloxane structure [33]. In Figure 2c, the ^29^Si nuclear magnetic resonance spectrum of the HSOS PSQ particles revealed an intense peak at −65.4 ppm for the fully polycondensed structure of [RSi(OSi)_3_] and a weak shoulder at a −75.8 ppm for an incomplete structure of [RSi(OSi)_2_OH] [34]. These peaks indicate that the samples contain a high amount of both Si–OR and Si–OH moieties from fully and incomplete condensed silanes, respectively. Figure 2d displays the N_2_ adsorption to desorption isothermal plot of the HSOS particles, which showed an H2-type hysteresis at high pressures (P/P_0_ of 0.8–1), thereby indicating that some macropores are present on the shell of the HSOS particles [35]. This finding suggested that the HSOS particles can be used as adsorbents given the presence of these macropores, even though the surface area of these spheres is not very large (125 m^2^/g).

### 3.2. Formation Mechanism of the HSOS Particles

**Figure 3:**
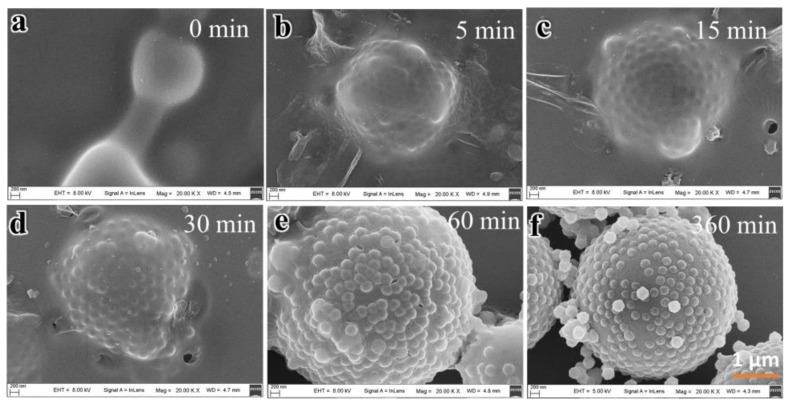
SEM images of the HSOS particles formed at different reaction times: (**a**) 0 min, (**b**) 5 min, (**c**) 15 min (**d**) 30 min, (**e**) 60 min, (**f**) 360 min.

To investigate the formation mechanism of the HSOS particles, the time-dependent imaging of the particles was conducted at regular time intervals during the reaction. In detail, a few suspension samples were taken out of the system for SEM characterization at different reaction times (Figure 3). At the beginning of the reaction (Figure 3a), the nanospheres containing PS appeared to first form with a smooth surface and then attached themselves to the microspheres in the final HSOS particles. When the reaction was performed for 5–30 min (Figure 3b–d), nanoprotrusions on the irregular microsphere surfaces were observed, and the particle sizes increased with a prolonged reaction time. In this process, the nanospheres stabilized by PVP and SLS are distributed at the oil–water interface (the surface of the oil droplet) via a hydrophilic–oleophilic interaction. The size of the microspheres grew from 1.2 µm to 3.4 µm as a result of the hydrolysis-polycondensation process of the silane precursor (oil droplet) with PVP and SLS co-stabilized PS nanoparticles as a solid Pickering emulsifier in the ice bath. At this stage, the silane precursor monomer remained incompletely polycondensed, and the emulsion droplets were not completely cured when they were taken out from the reaction system for the SEM analysis. Thus, the morphologies of the microspheres in Figure 3b–d are irregular and the nanoprotrusion seemed to have been grown from the microspheres. When reacted for 60 min (Figure 3e), regular raspberry-like particles could be clearly observed, and the morphology was found to be stable without drastic changes until reaction for 360 min (Figure 3f). At this stage, the polycondensation had reached a high level, and the core sphere was solidified with satellite shell particles on the surface that were fixed to form a regular raspberry structure.

### 3.3. Effect of Silane Precursor

**Figure 4:**
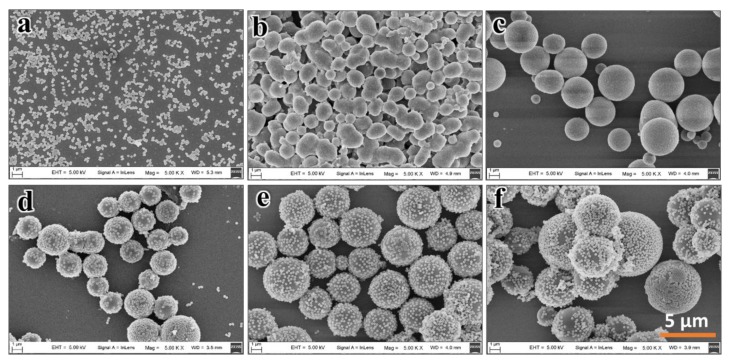
SEM images of HSOS particles with different mole ratios of methyltrimethoxysilane (MTMS) to 3-mercaptopropyltrimethoxysilane (SHTMS); (**a**) 0, (**b**) 2.5, (**c**) 5, (**d**) 10, (**e**) 15 and (**f**) 20, respectively.

The ratio of the MTMS to SHTMS plays an important role in the formation of the raspberry-like particles. Without MTMS, no microspheres were formed, and only irregular spheres with a diameter of 250 nm were observed (Figure 4a). When the mole ratio of MTMS to SHTMS was 2.5, the connected microspheres were observed with some free nanospheres randomly distributed between the microspheres (Figure 4b). When the mole ratio was increased to 5, a few nanoparticles and numerous microspheres with a smooth surface and without raspberry-like particles were observed (Figure 4c). When the mole ratio increased to 10, 15, and 20, raspberry-shaped microspheres formed (Figure 4d–f), and the average particle size of the microspheres gradually increased from 2.4 µm to 2.8 µm and 3.2 µm, but the diameter of the nanospheres remained at approximately 250 nm.

### 3.4. Effect of Catalyst

**Figure 5:**
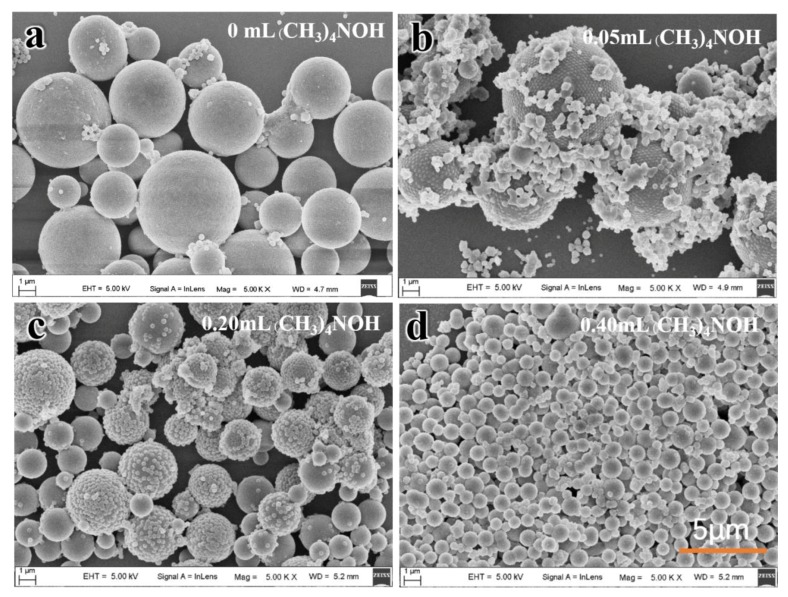
Effects of TMAH contents on the SEM images of HSOS particles; (**a**) 0 mL, (**b**) 0.05 mL, (**c**) 0.20 mL, (**d**) 0.40 mL.

TMAH, as an organic-based catalyst in the reaction, is essential to controlling the morphology of the HSOS particles. When the ammonium solution, rather than TMAH, was utilized, only smooth microspheres without raspberry-like microspheres were obtained (Figure 5a). When the amount of TMAH was 0.05 mL, large microspheres with aggregated irregular nanoparticles were obtained given the low reaction rate from the low concentration of the catalyst (Figure 5b). When the TMAH content was 0.1 mL, raspberry-like microspheres with nanospheres could be observed (Figure 1a). When the amount of the catalyst was further increased to 0.2 mL, the hydrolysis rate further increased, thereby resulting in irregular microspheres with slight agglomeration (Figure 5c). When the TMAH content was increased to 0.4 mL, no regular raspberry-like structures were formed. Only the microspheres and the nanospheres coexisted randomly (Figure 5d). A suitable concentration of the catalyst could be considered to cause the equilibrium of the hydrolytic condensation rate of the silane droplets along with the competitive role of the nanosphere emulsifier on the microspheres.

### 3.5. Effect of PS Latex

**Figure 6:**
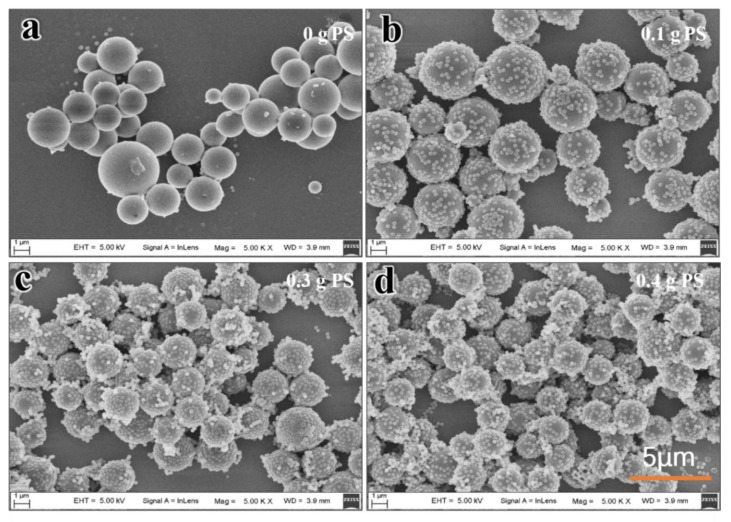
Effects of polystyrene (PS) latex contents on the SEM images of HSOS particles; (**a**) 0 g, (**b**) 0.1 g, (**c**) 0.3 g, (**d**) 0.4 g.

The presence of nanosized PS latex directly affected the formation of raspberry-like microspheres. When PS nanospheres were not added, only microsized spheres with smooth surfaces were observed without clear raspberry-like particles (Figure 6a). When the PS content was 1 g, the average size of the microsphere was approximately 3.2 µm, and the diameter of the nanosized spheres on the spherical structure was approximately 250 nm (Figure 6b). When the PS contents were 2, 3, and 4 g (Figure 1a and Figure 6c,d), the nanoparticle size remained consistent, whereas the average HSOS particle size decreased from approximately 2.4 µm to 2.3 µm and 2.1 µm. These decrease in particle sizes were attributed to the increase in the number of nanospheres. Additional PS particles exhibited added hindrance on the growth of the microsphere by covering the particle surface.

### 3.6. Effect of PVP and SLS

**Figure 7:**
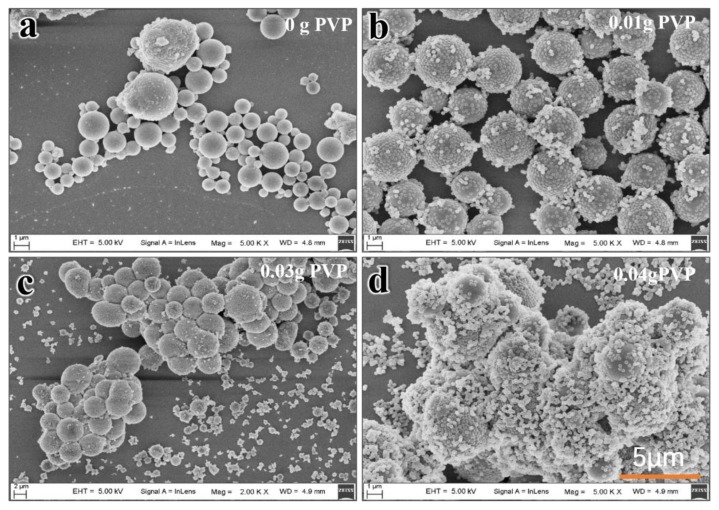
Effects of polyvinylpyrrolidone (PVP) contents on the SEM images of HSOS particles; (**a**) 0 g, (**b**) 0.01 g, (**c**) 0.03 g, (**d**) 0.04 g.

**Figure 8:**
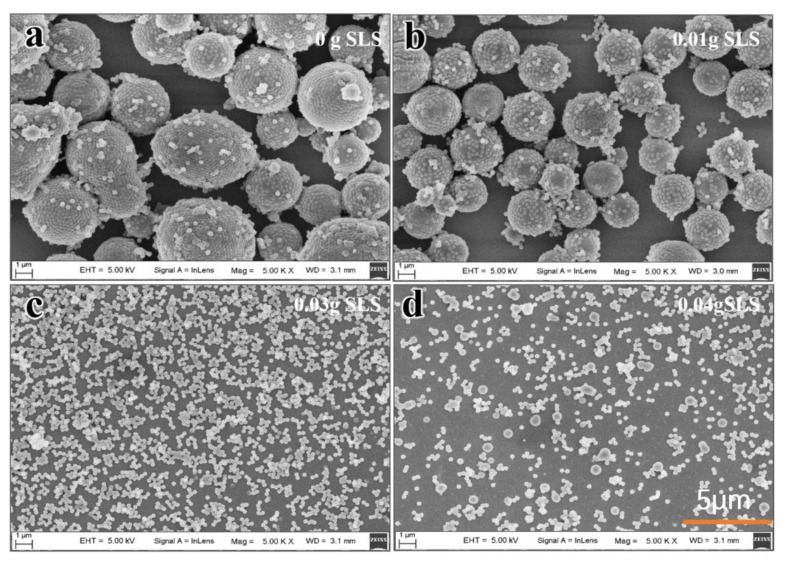
Effects of SLS contents on the SEM images of HSOS particles; (**a**) 0 g, (**b**) 0.01 g, (**c**) 0.03 g, (**d**) 0.04 g.

In this system, two surfactants of PVP and SLS significantly affected the formation process of the HSOS structure. In the beginning, the oil droplets were formed from the silane precursors of MTMS and SHTMS monomers under the synergistic effect of magnetic stirring and surfactant. Without PVP, nanoparticles were not attached to the surface of the microspheres, and microsized spheres exhibited a smooth surface with random sizes (Figure 7a). When the PVP contents were 0.01 and 0.02 g (corresponding to Figure 7b and Figure 1a), regular raspberry-like structures were formed with average particle sizes of 2.2 µm and 2.4 µm, respectively. When the PVP content was increased to 0.03 and 0.04 g, only irregular aggregated microsized particles and random free nanoparticles were observed (Figure 7c,d). The PVP adsorbed on the surface of the nanoparticles posed as the linker of nanoparticles and microspheres via its carbonyl group to form a hydrogen bond with a hydroxyl group on the surface of the PSQ core particle. Without using SLS (Figure 8a), irregular and large raspberry-like microspheres were formed with an average size of 4.0 µm. When 0.01 g or 0.02 g of SLS was utilized (Figure 8b and Figure 1b, respectively), regular raspberry-like microspheres were formed with sizes of 2.8 µm and 2.4 µm given the dispersion effect. When the amount of SLS was increased to 0.03 g (Figure 8c) and 0.04 g (Figure 8d), no regular microsized particles appeared. Thus, the hydrolysis–polycondensation rates on a silane oil droplet and on the PS nanospheres are balanced. Excessive SLS will break the balance to inhibit the formation of microspheres, thereby only accelerating the formation of PSQ on nanospheres.

### 3.7. Au@HSOSs for Catalyst Reduction of 4-NP

**Figure 9:**
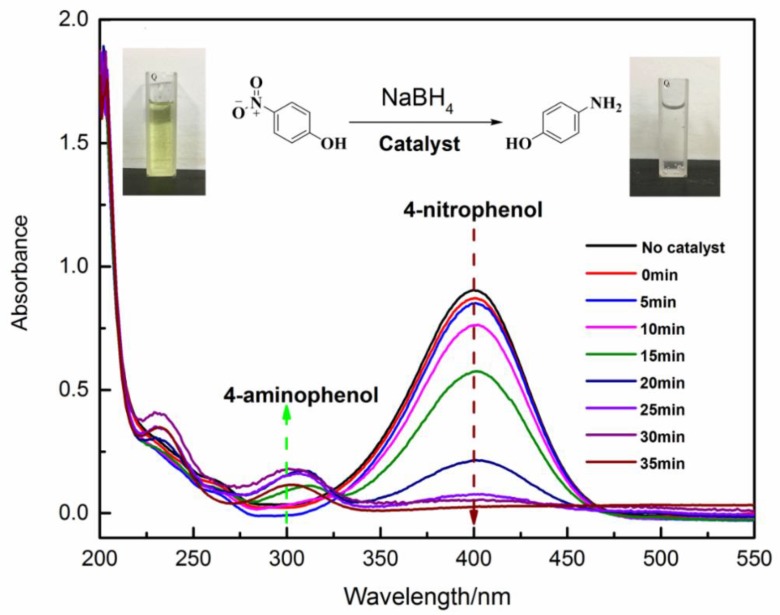
UV-Vis spectra of 4-nitrophenol reduction with Au/HSOSs as a catalyst. Insets are the reaction scheme, associated color change from bright yellow to colorless and the conversion of 4-nitrophenol graph in 35 min.

The new HSOS-structured particles were obtained by soaking the as-prepared raspberry-like particles in THF. The hollow structure combined with the mercaptopropyl groups made the PSQ particles suitable for capturing metal ions. Well-dispersed Au nanoparticles were immobilized on the HSOS microspheres initially via the coordination between metal ions and the coordinative SH–PSQ shells via an impregnation–reduction process followed by reduction with a NaBH4 aqueous solution. From the TEM images depicted in Figure 1g,f, the morphology and size did not exhibit an evident difference in comparison with the materials before Au loading. In Figure 1f,g, the immobilization of Au nanoparticles (2–5 nm) on the HSOS microspheres was clearly indicated by the dispersed black dots on the hollow nanospheres. In accordance with the ICP data, the Au content in the composite was 3.8 wt.%. The as-prepared Au@HSOSs were utilized as the catalyst to reduce 4-nitrophenol (4-NP) into 4-aminophenol (4-AP) in the presence of NaBH4. Without a catalyst, no evident color change occurred, thus indicating that 4-NP reduction did not occur. When Au/HOSOSs were used as catalysts, and the absorption intensity was at 400 nm, which is the characteristic peak for 4-NP, 4-NP decreased successively with time (Figure 9). A new peak at 300 nm gradually appeared, thereby indicating the production of 4-AP [28]. The complete conversion of 4-NP was confirmed by the color change in the system from bright yellow to colorless (inset of Figure 9). The recyclability of Au/HSOS was studied, and 86.9% of the 4-NP was reduced after five running uses, thus indicating that the presence of SOS support was sufficient to stabilize the catalytic nanoparticles by preventing their aggregation.

## 4. Conclusions

Here, we designed and fabricated novel raspberry-like PSQ particles with hollow-nanosphere-on-dense-microsphere structure via a facile and robust Pickering emulsion polymerization. For the controllable synthesis of the materials, the effects of the reaction conditions, including silane precursor, PS nanoparticles, TMAH, and two surfactants, on the morphology of the particles were systematically investigated. Via the time-dependent studies on the medium product of the formation process, the reaction mechanism was revealed to involve the formation of PSQ-coated nanospheres, followed by the appearance of microspheres surrounded by nanospheres. The resulting particles possessed concurrent features of active organic surfaces and hollow shells, thereby indicating that they could encapsulate guest molecules. As catalyst carriers, Au nanoparticles had been immobilized on the hollow nanospheres of the HSOS particles, thus demonstrating the high activity in the catalytic reduction of 4-NP. These HSOS particles exhibited the potential for catalytic applications, high-performance liquid chromatography, and drug delivery.
